# Scalp Acupuncture Enhances the Functional Connectivity of Visual and Cognitive-Motor Function Network of Patients with Acute Ischemic Stroke

**DOI:** 10.1155/2020/8836794

**Published:** 2020-12-02

**Authors:** Huacong Liu, Lanpin Chen, Guifeng Zhang, Yijing Jiang, Shanshan Qu, Songyan Liu, Yong Huang, Junqi Chen

**Affiliations:** ^1^School of Traditional Chinese Medicine, Southern Medical University, Guangzhou, Guangdong, China; ^2^Zhaoqing Medical College, Zhaoqing, Guangdong, China; ^3^Department of Rehabilitation Medicine, Rehabilitation Hospital, Fujian University of Traditional Chinese Medicine, Fuzhou, Fujian, China; ^4^Department of Neurology, China-Japan Union Hospital of Jilin University, Changchun, Jilin, China; ^5^Department of Rehabilitation Medicine, The Third Affiliated Hospital of Southern Medical University, Guangzhou, Guangdong, China

## Abstract

**Design:**

A parallel-group randomized controlled trial. *Participants*. 30 hemiplegic patients with middle cerebral artery acute infarction of the dominant hemisphere. *Interventions*. 30 patients were divided into 2 groups randomly. 15 patients in the treatment group (TG) were treated with ISSA, needling at the parietal midline (MS5) and left anterior/posterior parietal-temporal oblique lines (MS6 and MS7), combined with western routine treatment. While another 15 patients in the control group (CG) received routine treatment only. *Main Outcome Measures*. (1) Functional connectivity (FC): patients received brain scan using 3.0 T MRI after the treatment for 1 week. Based on the Matlab2012a platform, SPM12 software and DPABI software were used to process the scanning data and finally the functional connectivity of the brain was obtained. (2) National Institute of Health Stroke Scale (NIHSS) score.

**Results:**

The difference in the NIHSS score between the two groups of patients before and after treatment was statistically significant (tNIHSS = 2.225; PNIHSS = 0.038), indicating that TG had a better effect. Centered to the seed region of the left supplementary motor area (SMA) (−5.32, 4.85, 61.38), FC increased at the left middle cerebellar peduncle, left cerebellum posterior lobe (uvula and declive), vermis, fusiform gyrus, lingual gyrus, inferior occipital gyrus, calcarine, cuneus, precuneus, BA7, BA18 and BA19, etc. Centered to the seed region of the left parahippocampal gyrus (PG) (−21.17, −15.95, −20.70), FC increased at the left precuneus, inside-paracingulate, inferior parietal gyrus, paracentral lobule, BA5, BA6, BA7, and BA40, right median cingulate, precuneus, BA19, BA23, and BA31, etc.

**Conclusions:**

It is indicated that ISSA can regulate the brain functional connection in patients with middle cerebral artery acute infarction in the dominant hemisphere and specifically strengthen the connections between visual, cognitive, motor control, and planning-related brain regions, which may be related to the recovery of movement in the mechanism. This trial is registered with ChiCTR-IOR-15007672.

## 1. Introduction

Characterized by high morbidity, disability, and mortality rate, stroke has been ranked as the leading cause of motor disability across the world. Ischemic stroke is the most common type of stroke accounting for 85% of the total. The Global Burden of Disease [1] showed that the incidence of ischemic stroke in China was 201.74/100,000 in 2019 and the disability-adjusted life year as high as 1504/100,0000 [1].

The popularization of mature and appropriate treatment methods such as thrombolysis and thrombectomy can recanalize the patient's blood vessels in time which may turn the crisis into safety and improve the AIS's survival rate [2]. However, the high disability rate of the stroke patients will still remain the same. Both the stroke patients who missed the right time for thrombolysis and thrombectomy and those with contraindications need to begin rehabilitation treatment as early as possible to gain a better recovery [3].

Acupuncture is one of the traditional rehabilitation techniques. A relevant meta-analysis found that acupuncture can improve the motor function of the defect in acute stroke patients without obvious serious adverse events [4]. Among them, the ISSA therapy has been used in the clinical treatment of AIS by stimulating specific areas of the scalp [5]. However, modern medicine and rehabilitation training are mainly used in the acute stage of ischemic stroke at present. To further promote ISSA, the complementary therapy, the related mechanism needs a deeper study. Translational medicine believes that the mechanism research can provide a reference for clinical treatment [6]. Hence, investigating the mechanism of the specific effect of ISSA on the central nervous system in vivo may help introduce an effective method to treat brain-derived diseases.

In recent years, the development of medical imaging has provided a new means for the study of acupoint specificity [7, 8] and acupuncture mechanisms such as using electroencephalogram, magnetoencephalogram, and magnetic resonance to observe the changes in the brain structure and function. Acupuncture's imaging studies mainly focus on the effects of acupoints on the brain and the brain mechanism of treating suitable diseases by acupuncture, which includes specific research on acupoint, research in the basic factors of acupuncture, and mechanism research of treating diseases. Rs-fMRI is an important method to study the therapeutic mechanism of acupuncture in the treatment of diseases, especially to analyze the FC among different brain regions.

FC analyzes the functional connectivity intensity between brain regions by detecting the temporal correlations of blood oxygen level-dependent (BOLD) signals between spatially distant brain regions. FC obtains the changes in the overall brain functions of stroke patients, which is regarded as one of the hot spots in the study of mechanism of the therapeutic effect of acupuncture. Zhang et al. [9] believed that the possible mechanism of acupuncture regulating effect on the default mode network (DMN) in stroke patients could be explained the cognitive ability and motor function recovery. And, Wu et al. [10] pointed out that acupuncture led to a significant structural reorganization of the frontal lobe and DMN regions, which may be the potential mechanism of acupuncture to improve motor and cognitive recovery. However, the Rs-fMRI of ISSA in the treatment of AIS has not been reported. Our previous study showed that ISSA could specifically strengthen the connections among the brain regions involved in the regulation of cognitive and executive brain networks and the adjacent brain regions in healthy middle-aged and elderly adults [11]. Combined with the above literature, we hypothesized that ISSA could specifically adjust cognitive and executive brain networks' regulatory systems in the AIS patients.

Therefore, in order to further clarify the specific effect of ISSA on brain functional connectivity in AIS patients, this study observes the specific changes of brain functional connectivity in AIS patients after ISSA intervention by Rs-fMRI besides the observation of its curative effect.

## 2. Methods

### 2.1. Design

The study was a parallel-group randomized controlled trial, approved by the Ethics Committee of the China-Japan Union Hospital at Jilin University, on July 18, 2016 (approval no. 2016ks043), and has been registered at the Chinese Clinical Trial Registry (registration no. ChiCTR-IOR-15007672). Based on the complexity and particularity of fMRI image acquisition and data statistics of stroke patients, this paper estimates the sample size of the fMRI study on the acupuncture effect mechanism. Referring to Desmond et al. [11] and the imaging literature of acupuncture improving cognitive function or motor function of stroke patients at home and abroad [12, 13] and considering the 20% drop-off rate, 15 patients in each group were included in this study. In this study, patients were randomly assigned by using the random number table method, and the proportion was 1 : 1. The study separated the researchers responsible for generating randomized protocols, assigning protocols, recruiting patients, and evaluating efficacy and statistical analysis.

### 2.2. Participants

Thirty patients with acute infarction in the middle cerebral artery supply area in the dominant hemisphere were recruited from January 2017 to December 2017. The patients were randomly assigned into the TG of ISSA (15) and the CG of routine treatment (15).

The diagnostic criteria: the researchers adopt the relevant standards of the Guidelines for the Diagnosis and Treatment of Acute Ischemic Stroke 2014 [14] issued by the Chinese Medical Association Neurology Branch and the Chinese Medical Association Neurology Branch Cerebrovascular Disease Group in 2014. The inclusion criteria are as follows: patients (1) with typical lopsided motor and sensory disorders symptoms were diagnosed with AIS in the left middle cerebral artery blood supply area through the examination of MRI or computed tomography; (2) in stable condition; (3) whose age between 40 and 70 years and any gender; (4) who received western essential treatment (except the drugs for promoting collateral circulation such as urinary kallidinogenase and butylphthalide); (5) with right handedness; (6) who signed the informed consent and voluntarily accepted the test. The exclusion criteria are as follows: (1) stroke course longer than 72 hours; (2) cerebrovascular pathological variation; (3) patients with other heart, kidney, and liver diseases and tumors that affect the test results; (4) consciousness disorder, sensory aphasia/mixed aphasia, or psychiatric history of claustrophobia and dementia, which affects the communication and operation during the experiment; (5) patients who had a history of acupuncture in the past 1 month; (6) patients who received transcranial magnetic stimulation, or transcranial direct current therapy, or electroencephalography biofeedback therapy at the same time; (7) acupuncture contraindications such as hemophilia. Suspension and shedding criteria are as follows: (1) patient's condition suddenly worsened during the observation period and could not continue to be observed; (2) patients who had not completed the prescribed course of treatment or whose data were incomplete.

### 2.3. Intervention

#### 2.3.1. Conventional Methods: Modern Medicine

The patients received antiplatelet, lipid-lowering and plaque-fixed, temperature, blood pressure, and blood glucose controls, nutritional support, and other treatments according to the needs of the disease.

#### 2.3.2. ISSA Treatment

According to the International Standardization Scheme for Acupuncture Points of the Scalp [15–17], we selected the ISSA lines MS5, left MS6, and MS7 for the treatment. Refer to Appendix 1 for acupoint location. After routine disinfection, stainless steel needles (0.3 mm × 40 mm, Hwato, Suzhou, China^a^) were inserted into the depth of 30 mm at the angles of 15–30° with respect to the bottom of the epicranial aponeurosis. After Deqi, the needles were twisted at a frequency of 200 turns per minute, causing a distending sensation. Twisting was repeated at 10-minute intervals during the 30-minute period. Two times a day for six days as one course, the trial was finished after a course of treatment.

Patients of two groups were treated with routine medicine. TG patients were also treated by the same doctor who has obtained the license of the medicine practitioner and has been engaged in acupuncture clinical work for more than 3 years.

## 3. Main Outcome Measures and Data Analysis

### 3.1. FC

Brain scans were acquired using the MRI system (Siemens 3.0T, Siemens Healthineers, Germany^b^) before and after the treatment course. We conducted T1-weighted magnetization-prepared rapid acquisition of gradient-echo and fMRI-BOLD echo-planar images. Scanning precautions and the fMRI-BOLD scanning parameters are given in Appendix 2. Data processing included data pre-processing, nuisance covariates regressing analyzing for consistency with the whole brain data, and the statistical model was established and statistical parameter diagram was obtained. Among them, the researchers selected 116 brain regions in the AAL [18, 19] as seed regions. All seed regions were analyzed for consistency with the whole brain data to calculate the FC values of different seed regions in the two groups of patients. Refer to Appendix 3 for detailed statistical process.

### 3.2. NIHSS Scores and Its Subitem Scores Related to Motor Function

The NIHSS score was used to evaluate the neurological deficits of patients. Its subitem scores related to motor function were also the observation indicators of this paper. NIHSS score and motor function score of both the groups were acquired before and after the treatment course. Measurement data such as the NIHSS score were analyzed by *T* test, while count datasets such as gender were analyzed by the *χ*^2^ test. Statistical analysis was performed using SPSS 20.0 statistical software.

## 4. Results

### 4.1. General Information

**Figure 1:**
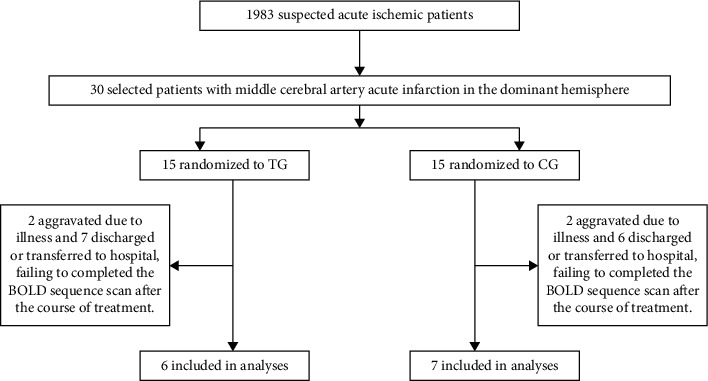
Inclusion situation of patients.

Overall, 17 patients discontinued the study prematurely and 13 patients were included in the analyzes (TG = 6; CG = 7) (Figure 1). The general information and clinical efficacy data of all 30 patients were retained. This study compares the gender, age, duration, systolic blood pressure, and diastolic blood pressure of only the patients who completed the BOLD series of scans in the two groups. The difference was not statistically significant (*P* > 0.05) and comparable (Table 1).

### 4.2. Comparison of NIHSS Scores and Motor Function Scores between the Two Groups before and after Treatment

Compared to the NIHSS scores and motor function scores in NIHSS before and after treatment of the two intragroups, there were statistical differences, which indicated that 2 kinds of treatment had a certain effect in treating AIS and improving the motor function. Comparison of the NIHSS scores and motor function scores before and after treatment of the TG with the CG showed there was not any statistical difference. Comparison of the differences of NIHSS scores and motor function scores before and after treatment of the 2 groups showed that the former had a statistical difference, while the latter had no statistical difference. The results indicated that the complementary ISSA might have a better effect than routine therapy (Tables 2 and 3).

### 4.3. FC Difference after Intervention with ISSA

**Figure 2:**
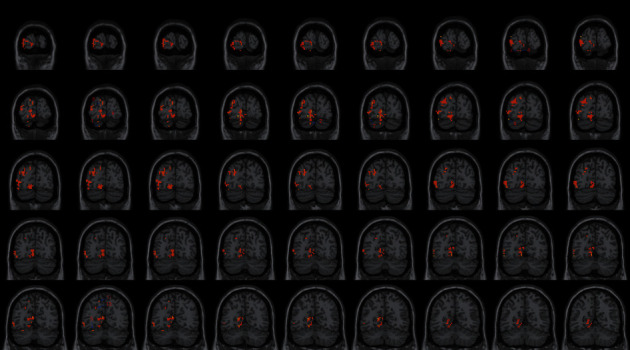
With the left SMA as the seed region, the international standard ISSA induces specific changes in the brain region for FC (coronal position).

**Figure 3:**
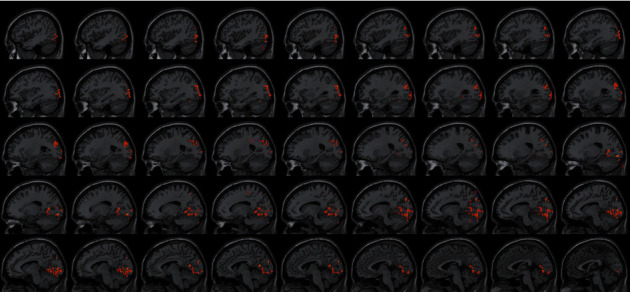
With the left PG as the seed region, the international standard ISSA induces specific changes in the brain region for FC (coronal position).

**Figure 4:**
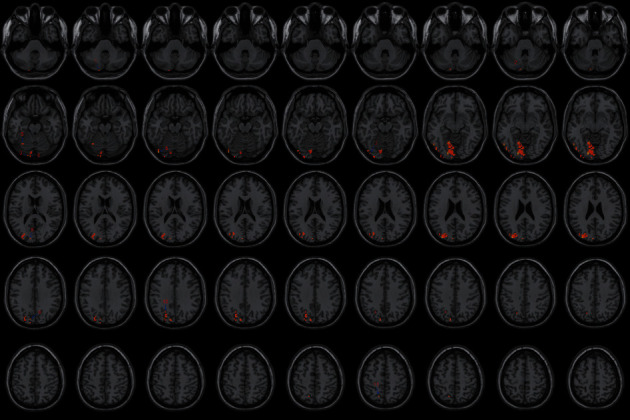
With the left SMA as the seed region, the international standard ISSA induces specific changes in the brain region for FC (horizontal position).

**Figure 5:**
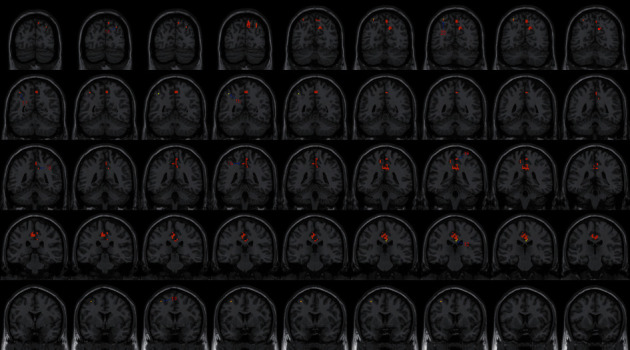
With the left SMA as the seed region, the international standard ISSA induces specific changes in the brain region for FC (sagittal position).

**Figure 6:**
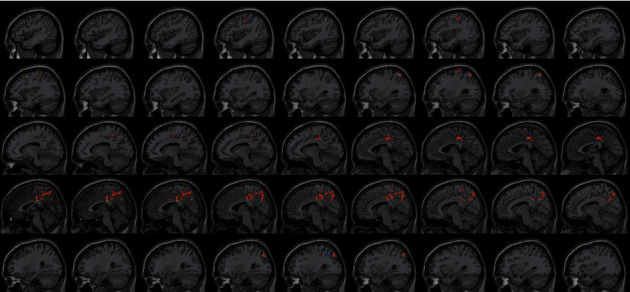
With the left PG as the seed region, the international standard ISSA induces specific changes in the brain region for FC (sagittal position).

**Figure 7:**
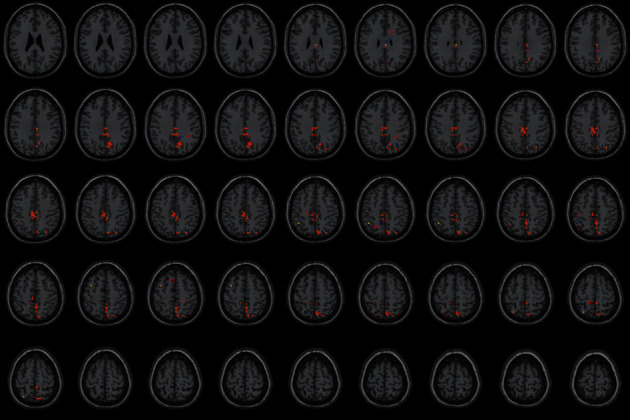
With the left PG as the seed region, the international standard ISSA induces specific changes in the brain region for FC (horizontal position). Note: the numbers represent different brain regions for FC. (1) Left cerebellum posterior lobe (uvula), (2) left cerebellum posterior lobe (declive), (3) left fusiform gyrus, (4) left lingual gyrus, (5) declive of the posterior left cerebellar lobe and the superior vermis, (6) left BA18, (7) left inferior occipital gyrus, (8) left cuneus (9) left BA19, (10) left precuneus, (11) left BA7, (12) right BA23 and median cingulate, (13) right median cingulate and inside-paracingulate (BA31), (14) right precuneus and BA19, (15) left BA40, (16) left precuneus and inside-paracingulate, (17) left inferior parietal gyrus, (18) left paracentral lobule, BA5, and inside-paracingulate, (19) left BA6, (20) left superior parietal gyrus.

Centered to the seed region of the left SMA (−5.32, 4. 85, 61.38), FC increased at the left precuneus extending to left cuneus and BA19, left lingual gyrus extending to calcarine, and also increased at the left middle cerebellar peduncle, left cerebellum posterior lobe (uvula and declive), vermis, fusiform gyrus, inferior occipital gyrus, cuneus, precuneus, BA7, BA18, and BA19, etc. Centered to the seed region of the left PG (−21.17, −15.95, −20.70), FC increased at the right median cingulate and inside-paracingulate (BA23) extending to precuneus, also increased at the left precuneus, inside-paracingulate, inferior parietal gyrus, paracentral lobule, BA5, BA6, BA7, and BA40, right BA19, BA23 and BA31, etc. (Table 4, Figures 2–7).

## 5. Discussion

The observation validated the efficacy of ISSA in the treatment of AIS patients, confirming that the ISSA combined with routine treatment could improve the neurological deficits and motor dysfunction in patients with AIS in the trial, which was consistent with those by Wang et al. [5]. Based on the effectiveness, we explored the specific effect of the ISSA on brain function networks in patients with dysfunction after AIS.

The SMA is an important key of the motor system, which plays a considerable role in the planning, execution, and control of complex motor functions [20]. SMA-proper is primarily connected with the premotor area, the primary motor cortex, and the cortical crista medullary tract and is functionally related mainly to the motor performance. While pre-SMA is more closely connected with the prefrontal cortex and other nonprimary motor cortices and is more prone to high-level motor control, such as movement planning and preparation [21–23]. SMA is the hinge between high-level regulation of movement and low-level execution. The results showed that the main brain regions with enhanced connectivity to the left SMA are mainly included:

1. Cerebellar-related areas: the uvula is closely connected with the primary and secondary vestibular neurons and plays a key role in the vestibular reflex [24]. Furthermore, it also receives proprioception, vestibular impulse, and visual, auditor, and tactile senses. Aside from that, it controls muscular tension and maintains body posture and balance by acting on the anterior horn of the spinal cord through the spinal tract. The declive can strengthen muscle tone and coordinate random motions. As for the middle cerebellar peduncle, a part of the cerebro-pons-cerebellar circuit ensures the accuracy and effectiveness of voluntary movements.
2. Visual-related brain regions: the visual cortex is the basis of vestibular-ocular reflex to control the visual motion. BA18 and cuneus (BA19) form the visual association cortex, while the calcarine is the main focus of the visual cortex, jointly involved in visual processing. Moreover, the cuneus is an important part of the visual cortex that regulates the vestibular-ocular reflex. The fusiform gyrus is the most important part of the “what” pathway for object category recognition [25, 26] and activated during visual orientation processing [27]. The lingual gyrus is involved in locating objects in space to determine their positions of objects relative to body parts, playing a role in vision-motion coordination.
3. The precuneus (BA7) is adjacent to the somatosensory cortex and visual cortex in the sagittal position and has three main task domains [28]. The anterior area is responsible for sensorimotor processing, the middle area is closely related to cognition, and the posterior area is involved in the visual tasks. The precuneus integrates sensorimotor through proprioceptive, visual, and other sensory inputs and participates in higher cognitive functions related to motor intention or early motor planning. In addition to the cerebellum-related areas of motor coordination and control, SMA is also related to the visual-cognitive-motor control.

The PG is an advanced center of cognition and emotion. It includes many complex functions, such as memory, sensory representation, spatial orientation, and object recognition [29, 30]. The study found that the brain regions with significant connections to the left PG are mainly included:

1. Sensorimotor network (SMN) related brain regions: SMN is primarily concerned with motor planning, motor execution, and sensory processing and is responsible for sensorimotor integration [19, 31]. The anterior central gyrus is involved in motor planning and execution. BA6 plays a regulatory role in the coordination of combined movement and postural movement through its effects on muscular tension, posture maintenance, and motor coordination [32]. The anterior part of the paracentral lobule is correlated with the movement of the lower limbs, while the posterior part receives information of pain, temperature, touch, pressure, position, and motion perception of the opposite side. The connectivity enhancement of the superior parietal gyrus is related to the processing of visual spatial information, such as orientation [33]. The inferior parietal gyrus is involved in motor selection and motor planning. Its BA40 belongs to the secondary somatosensory cortex, which responds to somatic stimulation and is regarded as the application center, mainly responsible for fine motor coordination [34]. The precuneus is an important brain area of SMN and plays an important role in sensorimotor integration.
2. Visual and tactile brain regions: BA19 is involved in visual processing. BA5 and BA7 form the somatosensory junction cortex that integrates various senses such as tactile and pressure or utilizes previously stored sensory experience.
3. Brain regions correlated with cognitive function: the precuneus is involved in many advanced cognitive functions as well as the integration of sensorimotor. The cingulate cortex is closely related to cognition and default network. The inside-paracingulate (dorsal anterior cingulate gyrus), which is part of the executive control network, has strong bilateral connections with the lateral prefrontal, parietal, anterior motor, and SMA, responsible for cognitive function.
4. Visual motor control area: the median cingulate (BA24), also known as cingulate motor area (CMA), is an advanced neural network regulatory executive function. BA24 has projections into the anterior part of the SMA, the primary motor cortex, the motor thalamus, and the spinal cord [35, 36]. Studies have shown that CMA neurons play a vital part in the preparation and execution of visually guided arm movements [37]. So, the connection-enhanced cingulate gyrus is associated not only with cognition but also with visual-motor control.

It showed that ISSA may stimulate the motor cortex of the brain by promoting the integration of perception and sensory information and establish the connection with cognition.

The neural process by which the brain controls spontaneous movement can be divided into three stages [38]: firstly, unified performance of perception happens through sensory processes; secondly, cognitive processes can be analyzed by the internal reference system which determines how to deal with spontaneous movement; thirdly, spontaneous movement occurs when the brain chooses a motion plan and carries it out. The intrinsic reference model contains all the sensory and motor information which are needed to perform a particular motion. The cerebellum captures and stores internal models to help with motor control. In combination with the above results, the connections between visual-related brain regions and motor-related decision-making brain regions were enhanced. The same went for the connections between cognitive-related brain regions and sensory-motor-related brain regions. The two enhancements were in the early stages of spontaneous movement controlled by the brain. In other words, it was in the stage of transition from the sensory process to the cognitive process. The subjects of this study were patients with hemiplegia and sensory impairment. After treatment, the motor function of the two groups improved, but the difference between the two groups was not significant. On the contrary, there was a significant difference in the total score of NIHSS. The improvement of the nerve function was more significant in the treatment group. We speculate that ISSA can promote the early recovery of sensation and cognition. This not only affects the brain's early control of spontaneous movement but also lays a foundation for further recovery of motor function and plays a positive role in promoting the recovery of other dysfunction. However, there is no relevant clinical evidence to support the improvement of sensory and cognitive impairment, which needs to be further explored in the future by increasing the sample size.

### 5.1. Study Limitations

There were some shortcomings in the lack of observed sample size in this study: the subject of study was limited to the patients with ischemic stroke in the left middle cerebral artery, with non-life-threatening acute phase; being required to complete 6 days of ISSA treatment; received 2 times of fMRI scans before and after treatment. Therefore, only a small number of samples was included, which needed further study.

## 6. Conclusion

It was preliminarily found that, in the treatment for the patients with middle cerebral artery acute infarction in the dominant hemisphere, the application of ISSA could specifically strengthen the connections between visual, cognitive, motor control, and planning-related brain regions, which might relate to the recovery of voluntary movement.

**Appendix 1 .**

MS5 is located at the mid-sagittal line between Baihui (DU20) and Qianding (DU21), which are, respectively, located at 5 cun and 3.5 cun posterior to the midpoint of the anterior hairline. MS6 is located at the line joining EX-HN1(A), which is 1 cun anterior to DU20, and Xuanli (GB6), which is three quarters down the side of the hairline between Touwei (ST8) (0.5 cun above the corner of the hairline) and Qubin (GB7) (located at the vertical line from the hairline anterior to the ear to the level of the apex of the ear). MS7 is located at the line joining DU20 and GB7.

**Appendix 2 .**

Each participant was laid supine on the examination bed with their head fixed in a coil. The researchers reduced the influence of visual and auditory stimulation by fitting the participants with eye masks and earplugs. The subjects were asked to avoid any mental activity. A single provocation echo-planar imaging sequence is in 8 mins, the repetition time is 2000 ms, echo time = 30 ms, flip angle = 90°, slice thickness = 3.5 mm, gap = 0.7 mm, voxel = 3.5 mm × 3.5 mm × 3.5 mm, field of view = 224 mm × 224 mm, phases per location = 240, matrix = 64 × 64, and number of slices = 37.

**Appendix 3 .**

Data were preprocessed using statistical parametric mapping 12 software (https://www.fil.ion.ucl.ac.uk/spm/) and DPABI software (http://rfmri.org/dpabi) in Matlab 2012a. Preprocessing included slice time correction, motion correction, normalising, nuisance covariates regression (white matter and cerebrospinal fluid), detrending, and filtering. Then, the researcher selected 116 brain regions in the AAL as seed regions. All seed regions were analyzed for consistency with the whole brain data to calculate the FC values of different seed regions in the two groups of patients. Using the DPABI software, with gender, age, last scanning time, systolic blood pressure, diastolic blood pressure, and head movements as covariates, two sets of abovementioned FC data were analyzed by two independent sample *t* tests to obtain statistical parameter maps. The statistical parameter map was identified and corrected to obtain the anatomical position and activation intensity of the brain region with significant difference in two groups' FC values of the different seed regions (TFCE, number of permutations = 5000, two tailed). Significant postintervention changes in functional connectivity based on anatomical knowledge and clinical experience were verified by experienced neurologists.

## Figures and Tables

**Table 1:** General information of patients.

Group	Case (*n*)	Gender (case)		Age (year)	Time between 2 scans (day)	Systolic pressure (mmHg)	Diastolic pressure (mmHg)
		Male	Female				
Treatment	6	4	2	59.67 ± 2.50	9.50 ± 1.05	143.00 ± 10.00	80.33 ± 10.23
Control	7	5	2	64.43 ± 7.37	8.79 ± 1.78	143.87 ± 9.61	88.57 ± 11.65
Statistics		*χ * ^2^ = 0.26		*t* = −1.50	*t* = 0.86	*t* = −0.15	*t* = −1.31
*P*		0.61		0.16	0.41	0.88	0.22

Note: time between 2 scans indicated the interval before and after treatment

**Table 2:** The changes of NIHSS scores in the two groups before and after treatment.

Group	Cases (*n*)	Before treatment	After treatment	Difference of NIHSS score	*t*	*P*
Treatment	15	6.20 ± 4.86	3.00 ± 3.80	3.20 ± 2.46	5.05	<0.001
Control	15	4.47 ± 4.55	2.80 ± 3.93	1.67 ± 1.05	6.17	<0.001
*t*		1.01	0.14	2.23	—	—
*P*		0.32	0.89	0.04	—	—

**Table 3:** The changes motor function scores in NIHSS in the two groups before and after treatment.

Group	Cases (*n*)	Before treatment	After treatment	Difference of motor function score	*t*	*P*
Treatment	15	2.93 ± 2.58	1.53 ± 2.33	1.40 ± 1.24	4.37	<0.05
Control	15	2.20 ± 2.81	1.47 ± 2.03	0.73 ± 1.03	2.75	<0.05
*t*		0.75	0.08	1.60	—	—
*P*		0.46	0.93	0.12	—	—

**Table 4:** FC difference after intervention with the international standard ISSA.

Seed region	Effect	Brain region	MiNi coordinate			Intensity (*T* value)
			*X*	*Y*	*Z*	
Left SMA (−5.32, 4. 85, 61.38)	Enhanced	Left middle cerebellar pcduncle and left cerebellum posterior lobe (uvala)	−15	−90	−36	9.8703
		Left middle cerebellar pcduncle and left cerebellum posterior lobe (declive)	−12	−87	−30	7.7964
		Left fusiform gyrus	−18	−93	−21	8.6148
		Left lingual gyrus	−12	−93	−21	6.4835
		Left lingual gyrus and calcarine	−18	−87	−9	18.2954
		Left declive of the posterior left cerebellar lobe and the superior vermis	−30	−63	−21	8.9651
		Left BA18	−42	−84	−18	15.2362
		Left inferior occipital gyrus	−39	−87	−15	8.4304
		Left cuneus	−12	−90	18	7.6706
		Left cuneus and BA19	−6	−90	24	10.0833
		Left cuneus and BA19	−12	−90	33	9.7124
		Left precuneus, cuneus, and BA19	−12	−84	39	9.7976
		Left precuneus	−24	−63	36	5.7268
		Left precuneus	−21	−66	42	5.5054
		Left precuneus and BA7	−15	−63	54	10.1682

Left PG (−21.17 −15.95 −20.70)	Enhanced	Left BA40	−42	−51	48	19.0798
		Left precuneus and inside-paracingulate	−3	−42	48	5.0477
		Left inferior parietal gyrus	−39	−54	51	7.7319
		Left paracentral lobule, BA5, and inside paracingulate	−12	−39	54	7.4153
		Left BA6	−36	3	54	17.9918
		Left superior parietal gyrus (BA7)	−30	−63	60	14.3336
		Right median cingulated and inside-paracingulate(BA23), expanding to the precuneus	3	−24	30	22.9531
		Right median cingulated and inside-paracingulate (BA31)	9	−45	36	10.1911
		Right precuneus and BA19	30	−78	39	9.8502

Note: *X*, *Y*, and *Z* coordinates represent the left-right, anterior-posterior, and superior-inferior axes in the MNI space, respectively. MNI : Montreal Neurological Institute.

## Data Availability

The raw functional magnetic resonance imaging data used to support the findings of this study have not been made available because raw data involves personal privacy.

## Abbreviations

AIS: Acute ischemic stroke
BOLD: Blood oxygen level dependent
CMA: Cingulate motor area
CG: Control group
DMN: Default mode network
fMRI: Functional magnetic resonance imaging
FC: Functional connectivity
ISSA: International Standard Scalp Acupuncture
NIHSS: National Institute of Health Stroke Scale
PG: Parahippocampal gyrus
Rs-fMRI: Resting-state functional magnetic resonance imaging
SMN: Sensorimotor network
SMA: Supplementary motor area
TG: Treatment group.
